# Trend of antidepressants before, during, and after pregnancy across two decades—A population‐based study

**DOI:** 10.1002/brb3.1441

**Published:** 2019-10-15

**Authors:** Yuelian Sun, Julie Werenberg Dreier, Xiaoqin Liu, Katja Glejsted Ingstrup, Merete Lund Mægbæk, Trine Munk‐Olsen, Jakob Christensen

**Affiliations:** ^1^ Department of Neurology Aarhus University Hospital Aarhus Denmark; ^2^ Department of Economics and Business Economics National Centre for Register‐Based Research Aarhus University Aarhus Denmark

**Keywords:** antidepressants, depression, epidemiology, pregnancy

## Abstract

**Introduction:**

Factors that influence antidepressant (AD) prescription and use during pregnancy are multiple including, in particular, the balance between the potential risk of untreated depression and the potential risk of AD treatment. Surveillance of temporal trends of AD use might identify areas requiring further research. We studied the use of ADs before, during, and after pregnancy using national data across two decades in Denmark.

**Methods:**

We included 1,232,233 pregnancies leading to live birth in Denmark between 1 January 1997 and 31 December 2016. Information on redemption of AD prescriptions was obtained from the Danish National Prescription Register.

**Results:**

We identified 29,504 (2.4%) pregnancies having at least one AD prescription (96,232 AD prescriptions) during pregnancy. The majority redeemed more than one prescription (69.7%) often for a single kind of AD (83.5%), and in 94% of the AD‐exposed pregnancies, the estimated duration of treatment was 1 month or longer. Prescription of ADs during pregnancy increased steadily from 0.4% in 1997 to 4.6% in 2011, but decreased thereafter to 3.1% in 2016. The proportion of pregnancies with ADs in 2011 was 6.05‐fold higher than that in 1997. The temporal trends in AD prescription in the years before and after pregnancy were similar to the trend during pregnancy. The decreasing use of ADs during pregnancy after 2011 was mainly driven by a decrease in the use of selective serotonin reuptake inhibitors (SSRIs), especially citalopram, the main type of SSRIs used in Denmark.

**Conclusion:**

Prescription of ADs during pregnancy in Denmark increased steadily from 1997 to 2011 but decreased sharply thereafter. More research is needed to show whether the same trend exists in other populations, like women of reproductive age, men of reproductive age, and old people, and other countries. We also need to find explanation for the decreasing trend in recent years and potential risk for untreated depression.

## Graphical abstract

The proportion of pregnant women who redeemed antidepressants (AD) during pregnancy increased sixfold from 1997 to 2011, but decreased sharply thereafter—similar trends were found before and after pregnancy. The decrease in the use of AD after 2011 was mainly due to a decrease in the prescribing of serotonin reuptake inhibitors (SSRIs) and in particular citalopram, the main type of SSRI used in Denmark.

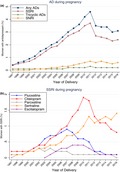

## SIGNIFICANT OUTCOMES

The proportion of pregnant women who redeemed antidepressants (ADs) during pregnancy increased six‐fold from 1997 to 2011, but decreased sharply thereafter—similar trends were found before and after pregnancy.

The decrease in the use of ADs after 2011 was mainly driven by a decrease in the prescribing of serotonin reuptake inhibitors (SSRIs) and in particular citalopram, the main type of SSRI used in Denmark.

## LIMITATIONS

Redeeming prescribed ADs during pregnancy is not always equivalent to the use of ADs, and exposure during pregnancy may thus be lower than estimated from pharmacy data.

## 1 INTRODUCTION

The prevalence of depression during pregnancy is substantial (Bennett, Einarson, Taddio, Koren, & Einarson, 2004; Gavin et al., 2005), and use of antidepressants (ADs) during pregnancy has increased in most developed countries in recent decades (Andrade et al., 2008; Jimenez‐Solem et al., 2013; Molenaar, Lambregtse‐van den Berg, & Bonsel, 2019). Antidepressant drug use during pregnancy increased from 2.0% of deliveries in 1996 to 7.6% of deliveries in 2004 and 2005 in the United States (Andrade et al., 2008). Dispensing rates of selective serotonin reuptake inhibitors (SSRIs) steadily increased in Netherland from 0.8% in 1999/2000 to 2.1% in 2013/2014 (Molenaar et al., 2019). In Denmark, the rate of AD exposure during pregnancy increased from 0.2% in 1997 to 3.2% in 2010 (Jimenez‐Solem et al., 2013).

Factors that influence AD use during pregnancy are multiple and include social, cultural, and economic factors, but in particular, the balance between the potential risk of AD treatment and untreated depression to fetus and pregnant women (Gentile, 2017; Gomez‐Lumbreras et al., 2019; Grigoriadis et al., 2013; Petersen, Gilbert, Evans, Man, & Nazareth, 2011; Prady, Hanlon, Fraser, & Mikocka‐Walus, 2018). Concerns about the safety of AD exposure to unborn babies are a major determinant of cessation of AD medication use during pregnancy (Petersen et al., 2011), although pregnancy can represent a time of increased vulnerability for the onset or return of depression (Bennett et al., 2004). The postpartum period is also a vulnerable time period with concerns of safety of medicine via breastfeeding and increasing demand for prescriptions of ADs (Munk‐Olsen, Gasse, & Laursen, 2012). Most studies have indicated that new types of ADs‐like selective serotonin reuptake inhibitors (SSRIs) are generally safe and not associated with birth defects or neurodevelopmental impairment (Petersen, Evans, Gilbert, Marston, & Nazareth, 2016; Prady et al., 2018), which might partly explain the increasing use of AD (and SSRIs in particular) during pregnancy in most countries. Side effects of AD use to the pregnant women are also important factors, which might affect prescriptions of ADs from physicians and adherence of AD use. Surveillance of temporal trends in AD use is basic and essential to research on health of pregnant women and unborn babies and might inform potential safety signals and identify areas requiring further research (Charlton et al., 2015).

In this study, we present the use of ADs in Denmark before, during, and after pregnancy between 1997 and 2016.

## 2 MATERIAL AND METHODS

### 2.1 Study population

We identified pregnancies leading to live birth between 1 January 1997 and 31 December 2016 in Denmark (*N* = 1,243,729) from the Danish Medical Birth Registry (Bliddal, Broe, Pottegard, Olsen, & Langhoff‐Roos, 2018; Knudsen & Olsen, 1998). We excluded pregnancies where the pregnant woman's identification number was missing (*n* = 9), pregnancies that had a missing value of gestational age (*n* = 11,363) or gestational age <20 weeks (*n* = 124), leaving 1,232,233 pregnancies in the study population. In Denmark, all residents are assigned a unique identification number recorded in the Danish Civil Registration System (Schmidt, Pedersen, & Sorensen, 2014), and this identification number enables linkage between the many nation‐wide registries, such as the Danish Medical Birth Registry and the Danish National Prescription Registry (Kildemoes, Sorensen, & Hallas, 2011) we used in this study. The study was a population‐based study and approved by the Danish Data Protection Agency.

### 2.2 Information on redeeming of ADs

Information on redeemed AD prescriptions was obtained from the Danish National Prescription Registry (Kildemoes et al., 2011), which contains data on all prescription medications dispensed from Danish community pharmacies since 1995. The registry has applied codes for medications using the anatomical therapeutic chemical (ATC) system. ADs were identified by the ATC code N06A. ADs were further categorized into the following groups: selective serotonin reuptake inhibitors (SSRIs), serotonin–norepinephrine reuptake inhibitors (SNRIs), tricyclic ADs (TCAs), and other ADs (ATC codes are found in Table S1). The register contains information on number of package of a medicine in a prescription and the number of defined daily dose (DDD) in one package. DDD is defined as the assumed average maintenance dose per day for a drug used for its main indication in adults (WHO, 2018).

### 2.3 Information on pregnancy

Information on gestational age and the age of pregnant women at the time of birth was obtained from the Danish Medical Birth Registry (Knudsen & Olsen, 1998). Information on gestational age was reported in days by the midwife attending the delivery using a mandatory coding sheet. Ultrasound measurements have been widely used to determine gestational age in nearly all pregnancies since 1995 (Jorgensen, 1999). Gestational age was used to estimate the time of conception by subtracting gestational age from the birth date. Information on diagnosis of depression and other psychiatric disorders before the time of delivery was obtained from the Danish Psychiatric Central Register, in which the International Classification of Diseases (ICD), 8th revision was used between 1966 and 1993 and 10th revision was used from 1994 onwards (Mors, Perto, & Mortensen, 2011; Munk‐Jorgensen & Mortensen, 1997). The codes we used to identify depression and other psychiatric disorders are shown in Table S2. To identify ADs that were prescribed before conception but may have been consumed by women in the early period of pregnancy, we defined the study period of pregnancy as the period from 1 month (30 days) before the estimated time of conception until birth. We also defined the two periods around pregnancy as 1 year (365 days) before and after pregnancy (1 year before pregnancy was defined as the period between 395 and 30 days before the conception). We used the common concept on periods of 1 year before and after pregnancy since we aimed to present temporal trend before and after pregnancy as well rather than comparing the exact prevalence of AD in the three periods.

### 2.4 Statistical analyses

For pregnancies with at least one AD prescription, we analyzed the number of AD prescription per pregnancy, the number of kind of AD per pregnancy, proportion of pregnancies with a type of AD (SSRIs, SNRIs, TCAs) or each specific AD, and the estimated duration that AD prescriptions during pregnancy might cover. If two or more kinds of ADs were prescribed on the same day, the prescription for each kind of AD was counted as a single prescription. We presented AD prescriptions with two kinds of ADs redeemed during pregnancy, which could be treatment with two kinds of AD or change from one kind of AD to another. We calculated the estimated duration that an AD prescription might cover by multiplying the number of redeemed packs with the number of defined daily doses (DDD) in one package. The total duration that AD prescriptions during pregnancy might cover was the summary of duration of all prescriptions during pregnancy. We presented duration for individual prescriptions and duration that all AD prescriptions might cover for pregnancies with one kind of AD during pregnancy. We presented proportion of pregnancies where women delivered at three age groups (<25, 25–34, 35+ years old) and proportion of pregnancies with a history of psychiatric disorders before the time of delivery (depression, other psychiatric disorders, or no psychiatric disorder) since the two factors could affect prescription and use of ADs.

We analyzed the temporal trend of overall AD prescriptions, and trends according to types of ADs, and specific AD during pregnancy. We analyzed the temporal trend of AD prescriptions in the 1 year before and 1 year after pregnancy. We further analyzed the trends in overall AD prescriptions according to pregnant women's age at the time of birth and the pregnant women's history of psychiatric disorders before the time of delivery dating back to 1969 (Mors et al., 2011). We analyzed the temporal trend of AD prescriptions during pregnancy by adjusting for the pregnant women's age at time of birth and the history of psychiatric disorders before the birth using a generalized linear model. We specified that the distribution of AD prescriptions is a Poisson distribution, the link function is logarithm, and women from different pregnancies are a cluster factor. The unit in the analyses was pregnancy, and one woman could have several pregnancies.

The analyses were conducted in STATA 15.1 (StataCorp LLC).

## 3 RESULTS

In the whole cohort, we identified 29,504 (2.4%) pregnancies where the pregnant women redeemed at least one AD prescription from 30 days before pregnancy to the date of the birth.

Among the 29,504 pregnancies where the pregnant women redeemed prescriptions for ADs during pregnancy, 30.3% redeemed one AD prescription and 69.7% redeemed two or more AD prescriptions including 50.6% with three or more AD prescriptions (Table 1). We identified 96,232 AD prescriptions in total during all pregnancies. Of all the pregnancies with AD prescriptions during pregnancy, 24,643 (83.5%) pregnant women had redeemed only one kind of AD, while 4,315 (14.6%) redeemed two kinds of ADs, and 546 (1.9%) redeemed three or more kinds of ADs. In total, 81.9% of pregnancies were exposed to SSRIs, 13.1% to SNRIs, and 6.4% to TCAs. Citalopram, sertraline, and fluoxetine were the main types of SSRIs prescribed during pregnancy (Table 1).

**Table 1:** Antidepressant (AD) prescriptions redeemed during pregnancy in Denmark between 1997 and 2016

Profiles	*N* = 29,504	
	No.	%
Number of AD prescription per pregnancy		
1	8,925	30.3
2	5,641	19.1
3	5,111	17.3
4	2,993	10.1
5	2,064	7.0
6	1,630	5.5
7+	3,140	10.6
Number of kind of AD per pregnancy		
1	24,643	83.5
2	4,315	14.6
3+	546	1.9
Type of and specific AD prescribed during pregnancy		
Tricyclic AD (nonselective monoamine reuptake inhibitors)	1,895	6.4
Selective serotonin reuptake inhibitors (SSRIs)	24,173	81.9
Zimeldine	0	0
Fluoxetine	5,117	17.3
Citalopram	10,168	34.5
Paroxetine	1,766	6.0
Sertraline	7,967	27
Alaproclate	0	0
Fluvoxamine	23	0.1
Etoperidone	0	0
Escitalopram	1,892	6.4
Serotonin–norepinephrine reuptake inhibitors (SNRIs)	3,857	13.1
Venlafaxine	3,230	10.9
Milnacipran	0	0
Duloxetine	679	2.3
Desvenlafaxine	0	0
Others	2,115	7.2
Nonselective monoamine oxidase inhibitors	NA	NA
Monoamine oxidase A inhibitors	13	0
Norepinephrine–dopamine reuptake inhibitors (NDRIs)	197	0.7

Note

NA: The number is <4 and not available according to data protection guidelines from Denmark Statistics.

Most AD prescriptions (88.5%) cover treatment for 1 month or longer. In 94% of the AD‐exposed pregnancies, the estimated duration of treatment was 1 month or longer (Table 2). Even among pregnancies with only one AD prescription, 84.7% would cover 1 month or longer (Table 2).

**Table 2:** Duration AD prescriptions during pregnancy might cover according to defined daily dose (DDD) of a drug for maintenance for its main indication

Duration AD prescriptions during pregnancy might cover	No.	%
All prescriptions (*N* = 96,232)		
<1 month	11,054	11.5
1 month	28,998	30.1
2 months	13,391	13.9
3 months	28,111	39.2
4–7 months	13,922	14.5
8+ months	756	0.8
All pregnancies with one kind of AD during pregnancy (*N* = 24,643)		
<1 month	1,478	6.0
1 month	3,483	14.1
2 months	2,105	8.5
3 months	3,476	14.1
4–7 months	4,591	18.6
8+ months	9,510	38.6
Pregnancies with one prescription of same AD		
<1 month	1,367	15.3
1 month	3,032	34.0
2 months	942	10.6
3 months	2,667	29.9
4–7 months	828	9.3
8+ months	89	1.0
Pregnancies with two prescriptions of same AD		
<1 month	106	2.1
1 month	356	7.2
2 months	984	19.9
3 months	347	7.0
4–7 months	2,159	43.8
8+ months	982	19.9
Pregnancies with three prescriptions of same AD		
<2 months	77	1.8
2 months	111	2.5
3 months	378	8.7
4–7 months	685	15.7
8+ months	3,115	71.3
Pregnancies with four and more prescriptions of same AD		
<2 months	23	0.3
2 months	68	1.1
3 months	84	1.3
4–7 months	919	14.3
8+ months	5,324	83.0

Note

One month is 28 days.

Among 4,315 pregnancies with two types of ADs during pregnancy, the most common combinations were fluoxetine and sertraline (11.0%), fluoxetine and citalopram (10.8%), citalopram and sertraline (8.6%), and sertraline and SNRIs (8.4%) (Table 3).

**Table 3:** Antidepressant (AD) prescriptions with two types of AD during pregnancy (*N* = 4,315)^a^

AD	Tricyclic AD		Fluoxetine		Citalopram		Paroxetine		Sertraline		Escitalopram		SNRIs		Other ADs		Total	
	No.	%	No.	%	No.	%	No.	%	No.	%	No.	%	No.	%	No.	%	No.	%
Tricyclic AD			49	1.1	89	2.1	21	0.5	103	2.4	18	0.4	62	1.4	34	0.8	451	10.5
Fluoxetine	49	1.1			464	10.8	201	4.7	474	11.0	108	2.5	240	5.6	120	2.8	1,657	38.4
Citalopram	89	2.1	464	10.8			93	2.2	370	8.6	283	6.6	217	5.0	223	5.2	1,739	40.3
Paroxetine	21	0.5	201	4.7	93	2.2			87	2.0	7	0.2	9	0.2	22	0.5	440	10.2
Sertraline	103	2.4	474	11.0	370	8.6	87	2.0			130	3.0	363	8.4	213	4.9	1,742	40.4
Escitalopram	18	0.4	108	2.5	283	6.6	7	0.2	130	3.0			38	0.9	39	0.9	623	14.4
SNRIs	62	1.4	240	5.6	217	5.0	9	0.2	363	8.4	38	0.9			116	2.7	1,078	25.0
Other ADs	34	0.8	120	2.8	223	5.2	22	0.5	213	4.9	39	0.9	116	2.7			778	18
Total	451	10.5	1,657	38.4	1,739	40.3	440	10.2	1,742	40.4	623	14.4	1,078	25.0	778	18		

Abbreviations: SNRIs, Serotonin–norepinephrine reuptake inhibitors; SSRIs, Selective serotonin reuptake inhibitors refer to fluoxetine, citalopram, paroxetine, sertraline, escitalopram in the table.

a The two types of AD could be a multitherapy of AD treatment or a change of AD from one type to another. Four pregnancies with a prescription of fluvoxamine during pregnancy were not included in the table because the numbers were too small according to data protection guidelines from Denmark Statistics.

The proportion of pregnant women who gave a birth at 35 years old and above was 12.7% in 1997 and 20.7% in 2016 (Table S3). The mean age of pregnant women at time of birth was 29.1 (standard deviation [*SD*]: 4.7) in 1997 and 30.3 (*SD*: 5.0) in 2016. The proportion of pregnant women who were diagnosed with depression and other psychiatric disorders before birth increased from 1997 to 2016 (Table S4).

**Figure 1:**
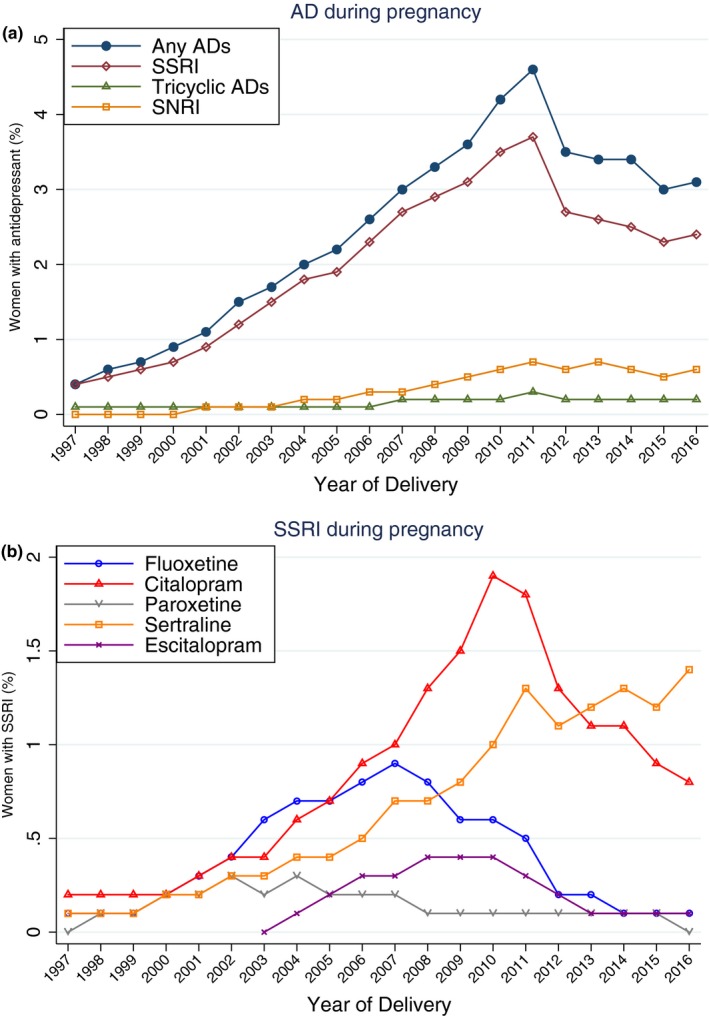
Trends in antidepressant prescriptions including types (upper panel) of and specific AD (bottom panel) during pregnancy in women who gave birth between 1997 and 2016 in Denmark

**Figure 2:**
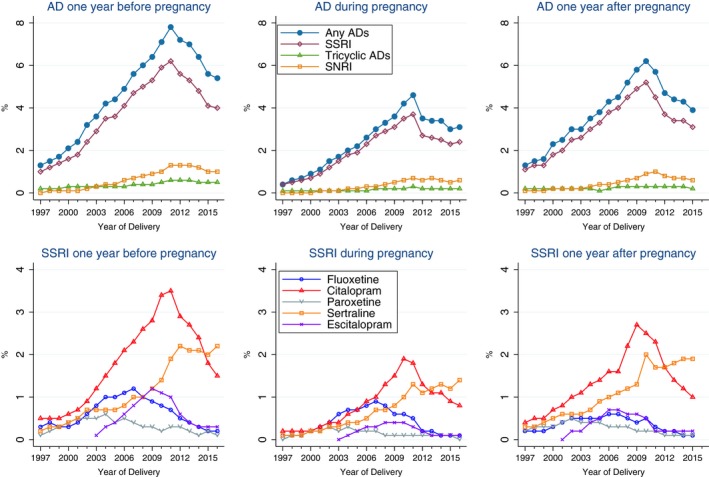
Trends in antidepressant prescriptions including types (upper panel) of and specific AD (bottom panels) before, during, and after pregnancy in women who gave birth between 1997 and 2016 in Denmark

The proportion of pregnancies with AD prescriptions rose steadily from 0.4% in 1997 to 4.6% in 2011, but decreased thereafter to 3.1% in 2016 (Figure 1). The decreasing use of ADs during pregnancy after 2011 was mainly driven by a decrease in use of SSRIs. Prescriptions of SNRIs also peaked in 2011 and slightly levelled off afterward. Among the individual SSRIs, prescription of citalopram and escitalopram decreased, and prescription of sertraline levelled off from 2011, while prescription of fluoxetine began to decrease from 2008 (Figure 1). Temporal trends in the prescription of ADs before and after pregnancy were similar to the pattern of AD prescription during pregnancy (Figures S1 and S2). The proportion of women who redeemed prescriptions for ADs in the year before pregnancy was 1.3%, 7.8%, and 5.4% for those delivered in 1997, 2011, and 2016, respectively. The proportion of women who redeemed prescriptions for ADs in the year after pregnancy was 1.3%, 5.7%, and 3.9% for those delivered in 1997, 2011, and 2015, respectively. The temporal trends of AD prescriptions before, during, and after pregnancy are shown in Figure 2. Information about AD prescriptions in the year after pregnancy among pregnancies delivered in 2016 is not shown in the figure since most of the women who gave birth in 2016 were not completely followed for 1 year since the prescription data were only available till the year 2016.

**Figure 3:**
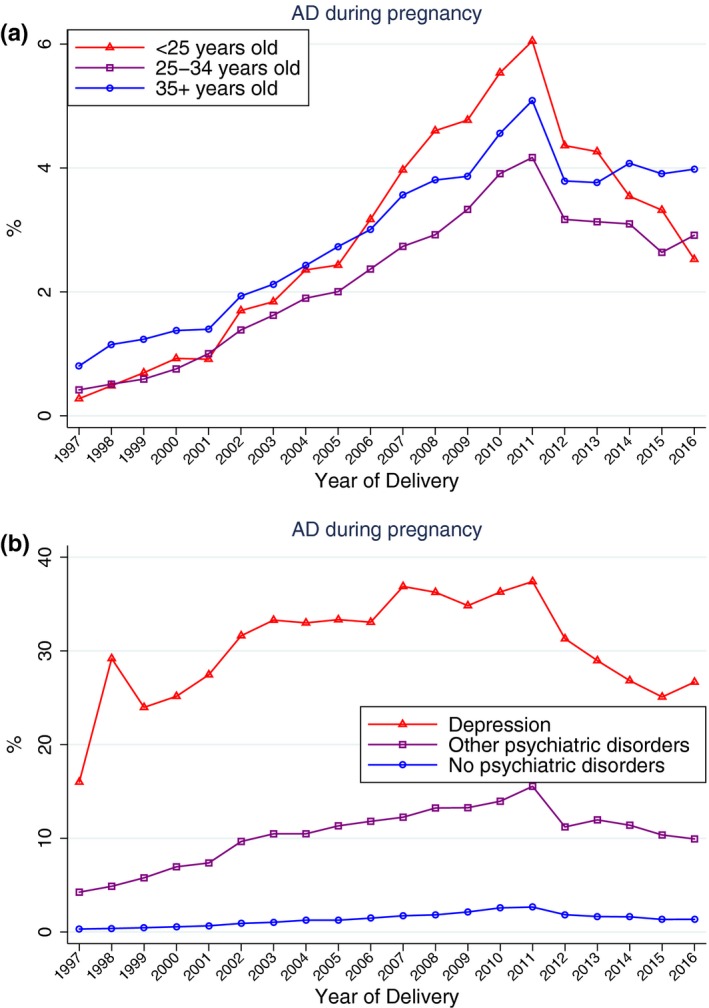
Trends in antidepressant prescriptions during pregnancy among women who gave birth in Denmark between 1997 and 2016 according to the age of the pregnant women (upper panel) and the history of psychiatric disorders before birth (bottom panel)

**Figure 4:**
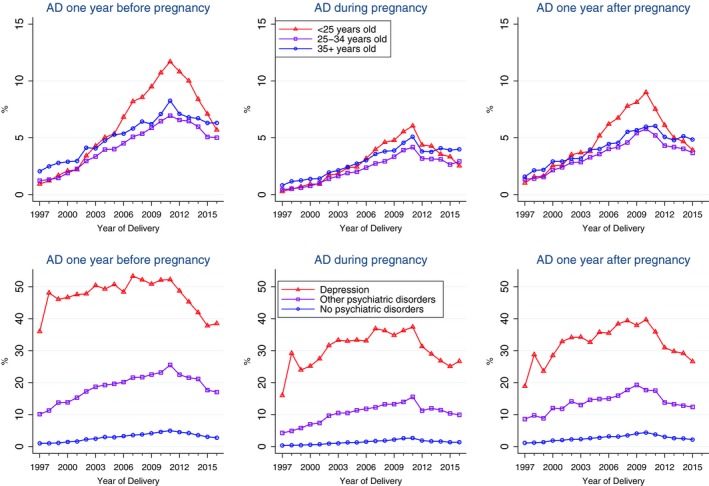
Trends in antidepressant prescriptions before, during, and after pregnancy among women who gave birth in Denmark between 1997 and 2016 according to the age of the pregnant women (upper panels) and the history of psychiatric disorders before birth (bottom panels)

**Figure 5:**
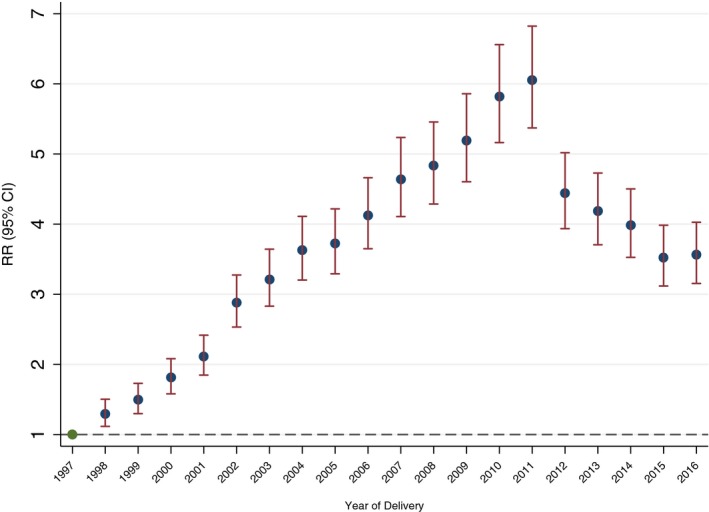
The risk ratio (RR) of redemption of AD prescriptions during pregnancy between 1998 and 2016 in Denmark compared with the prescription in 1997

Figure 3 shows the temporal trend in AD prescriptions during pregnancy according to the age at time of delivery and the history of psychiatric disorders of pregnant women. Redemption of AD prescriptions among women <25 years of age increased faster than among the other two groups of pregnant women (25–34 years of age and 35+ years of age), but all had a decrease in the use of ADs after 2011 (Figure 3). Temporal trends in AD prescriptions among pregnant women with and without a history of psychiatric disorders were different among the three groups, but all had a decrease in the use of ADs after 2011 (Figure 3). Temporal trends in the prescription of ADs in 1 year before and 1 year after pregnancy according to the age of the pregnant women and the history of psychiatric disorders before birth are shown in Figures S3 and S4. The temporal trends in antidepressant prescriptions before, during, and after pregnancy according to the age of the pregnant women and the history of psychiatric disorders before birth are shown in Figure 4. After adjusting for the age of women at the time of birth and the history of psychiatric disorders before birth, the proportion of prescriptions of ADs during pregnancy in 2011 was 6.05‐fold (95% CI: 5.37–6.82) higher compared with the proportions in 1997 (Figure 5).

## 4 DISCUSSION

Our study showed that 2.4% of all included pregnancies in Denmark during 1997 and 2016 had at least one AD prescription during pregnancy. In pregnancies with ADs, the majority redeemed more than one prescription (69.7%), most often for a single kind of AD (83.5%). Furthermore, in 94% of the AD‐exposed pregnancies, the estimated duration of treatment was 1 month or longer. SSRIs dominated in Denmark, and citalopram was the main type of SSRIs used in Denmark. The more impressive findings were that the proportion of pregnancies with AD prescriptions during pregnancy increased steadily from 1997 to 2011, but decreased sharply thereafter to the end of the study period in 2016. The decreasing trend in AD prescriptions in recent years was also found before and after pregnancy. The decrease in redeeming of AD prescriptions was driven mainly by a decrease in prescription of citalopram.

This was a population‐based study covering the entire population of pregnancies in Denmark over 20 years. Both the estimation of the duration of pregnancy (gestational age) and information on ADs were captured from nation‐wide registries, and these data are therefore not subject to recall bias. The quality of prescription data is also generally held to be high because of the Danish pharmacy reimbursement structure according to which all citizens receive reimbursement from the Danish regions. Reimbursement is automatically deducted from the price charged at the pharmacy. However, redeeming ADs is not always equivalent to using ADs. A study on the data quality of prescription registration in Denmark indicates that completeness of psychoanaleptics (N06) is 95.1% (Johannesdottir et al., 2012). A study in Denmark has shown that about 85% of people who were prescribed ADs took them regularly, which may also apply to AD use before women were aware of their pregnancy (Lewer, O'Reilly, Mojtabai, & Evans‐Lacko, 2015). A large proportion of pregnancies (50.6%) in our study had three or more AD prescriptions, and they were more likely to continue use AD during pregnancy.

The types of ADs used in pregnancy vary across countries (Abbing‐Karahagopian et al., 2014; Zoega et al., 2015). Since the 90s, use of SSRIs in Europe has increased while use of TCAs has remained stable or decreased except in Germany (Abbing‐Karahagopian et al., 2014). However, type of SSRIs varies across countries even among the homogeneous Scandinavian countries where citalopram was more often prescribed for pregnant women in Denmark, while sertraline dominated in Iceland and escitalopram dominated in Norway (Zoega et al., 2015). In pregnancies where the women filled two or more kinds of ADs during pregnancy, we did not separate the different situations like combination therapy with two or more kinds of ADs at the same time or change of AD from one kind to another (Benard‐Laribiere et al., 2018). This could be a group of interest for further studies.

Studies have shown that many factors including pharmaceutical expenditure, clinical factors, indications for use and patient, and doctor characteristics, can affect the prescription and use of Ads (Bauer et al., 2008; Gomez‐Lumbreras et al., 2019). In August 2011, the US Food and Drug Administration issued a safety warning concerning the safety of high doses of citalopram, as the administration of high doses was associated with cardiac corrected QT interval (QTc) prolongation according to findings from an unpublished randomized controlled trial (Howland, 2011). Citalopram was the most often prescribed AD to Danish pregnant women in 2011. This safety concern could, therefore, be one of the main explanations for the decreasing trend in AD use in Danish pregnant women after 2011, although the most recent guidelines published in 2010 for general practitioners in Denmark recommended that perinatal mental disorders should be pharmacologically treated (Munk‐Olsen et al., 2012; Zoega et al., 2015). It is still unknown whether the decreasing trend after 2011 is related to suboptimal treatment women with depression. Further studies are needed to determine time trends in other countries and especially whether the trends in AD prescriptions (e.g., of citalopram) show similar trends to those observed in this study.

In general, there was an increase in the proportion of prescriptions of ADs with increasing age of the pregnant woman (Abbing‐Karahagopian et al., 2014). Maternal age at birth increased slightly during the study period. The proportion of pregnant women who were diagnosed with depression or other psychiatric disorders before birth increased also with time, but both the age and history of depression or other psychiatric disorders could not explain the temporal trend of AD since the trend remained after adjustment for these factors.

Our study showed that the proportion of women (same as pregnancies) who redeemed AD prescriptions during pregnancy was lower than the proportion of women who redeemed AD prescriptions in the year before pregnancy and the year after pregnancy. We should be aware that the pregnancy period, which is usually around 9 or 10 months in this study since we included 1 month before pregnancy, is shorter than the other two periods, which are 1 year before and after pregnancy. It may slightly affect the proportion of women with AD prescription in these periods although it was unlikely to change the trend over time. Although the study population in the year before pregnancy comprised women who were not pregnant, they may not be representative of the general population of women of fertile age because pregnant women or women who are planning a pregnancy may overall be healthier than women of the same age who are not pregnant. Further studies are needed to determine whether the trend in AD prescriptions in the population of women of fertile age, men of fertile age, or old people follows the same pattern as that seen around pregnancy among pregnant women.

This study showed that the prevalence of AD use in 1 year after birth was higher than AD use during pregnancy, but lower than AD use in 1 year before pregnancy. The time after birth is a vulnerable time for new mothers and may herald the potential onset of new psychiatric conditions such as postpartum depression (Munk‐Olsen et al., 2016). At the same time, women are still cautious of taking medicine at the postpartum period especially when they are breastfeeding. A previous study showed that it takes about 2 years after birth to reach the prevalence level of AD use before pregnancy (Jimenez‐Solem et al., 2013). In UK and Wales, however, prescription of SSRI after birth was higher than prescription of SSRI before pregnancy (Charlton et al., 2015).

In conclusion, AD prescription before, during, and after pregnancy in Denmark rose steadily from 1997 and peaked in 2011 but has decreased sharply in recent years. The decrease in the trend of AD prescriptions was explained mainly by citalopram, the main type of SSRIs used in Denmark. More research is needed to show whether the same trend exists in other population, like women of reproductive age, men of reproductive age, and old people, and in other countries. More research is needed to find the explanation for the decreasing trend in recent years and the influence of the increasing and decreasing use of ADs during pregnancy for the health of pregnant women and their offspring.

## CONFLICT OF INTEREST

Dr. Christensen reported receiving honoraria from serving on the scientific advisory boards of, and giving lectures for, UCB Nordic and Eisai AB, as well as receiving travel funding from UCB Nordic. No other disclosures were reported.

## AUTHOR CONTRIBUTIONS

Dr. Sun had full access to all of the data in the study and takes responsibility for the integrity of the data and the accuracy of the data analysis. Sun, Dreier, Christensen, Liu, Ingstrup, Mægbæk, and Munk‐Olsen are involved in concept and design. Sun, Dreier, Christensen, Liu, Ingstrup, Mægbæk, and Munk‐Olsen are involved in acquisition, analysis, and interpretation of data. Sun drafted the manuscript, and Sun, Dreier, Christensen, Liu, Ingstrup, Mægbæk, and Munk‐Olsen performed critical revision of the manuscript for important intellectual content. Sun and Dreier statistically analyzed; Christensen obtained funding; and Christensen supervised.

## Data Availability

Data from the national prescription and medical birth registers used in this study are available and owned by the national health register holders in Denmark that provided permissions. Researchers can obtain access to individual‐level anonymized data via servers at Statistics Denmark and the Danish Health Data Authority.
