# Supplementary material for: Trend of antidepressants before, during, and after pregnancy across two decades—A population‐based study
Source: Brain Behav. 2019 Oct 15;9(11):e01441. doi: 10.1002/brb3.1441 (PMC6851806; doi:10.1002/brb3.1441)
Supplement: Supplementary file 1 [file BRB3-9-e01441-s001.docx]

Supplementary materials

**Title:** Trend of antidepressants before, during and after pregnancy across two decades – a population-based study

**Running title**: Trend of antidepressants around pregnancy

**Authors:** Yuelian Sun, ^1, 2^ Julie Werenberg Dreier, ^2^ Xiaoqin Liu, ^2^ Katja Glejsted Ingstrup, ^2^ Merete Lund Mægbæk, ^2^ Trine Munk-Olsen, ^2^ Jakob Christensen^1, 2^

**Affiliations**: ^1^ Department of Neurology, Aarhus University Hospital, Aarhus, Denmark;  ^2^National Centre for Register-Based Research, Department of Economics and Business Economics, Aarhus University, Denmark

**Corresponding author**: Yuelian Sun, Fuglesangs Allé 26, 8210 Aarhus V. Email: [ys@clin.au.dk](mailto:ys@clin.au.dk)

**Supplementary figures**

Supplementary Figure 1. Trends in antidepressant prescriptions including types (upper panel) of and specific AD (bottom panel) in one year before pregnancy in women who gave birth between 1997 and 2016 in Denmark


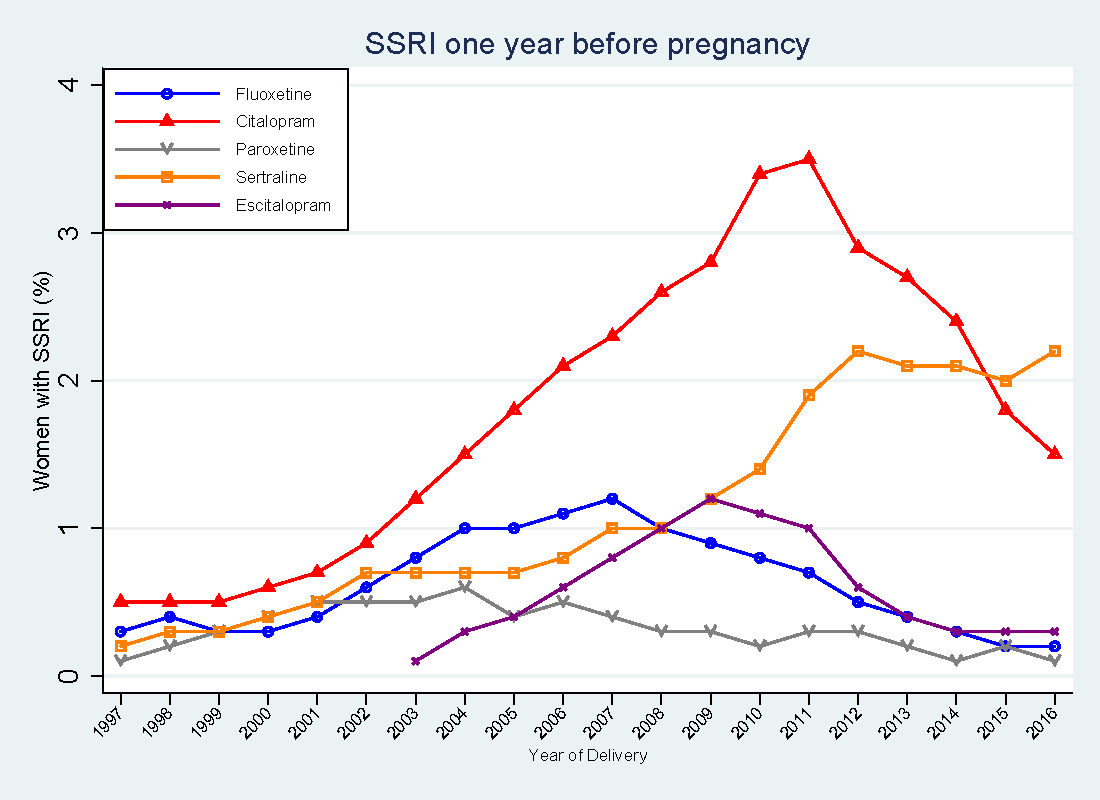


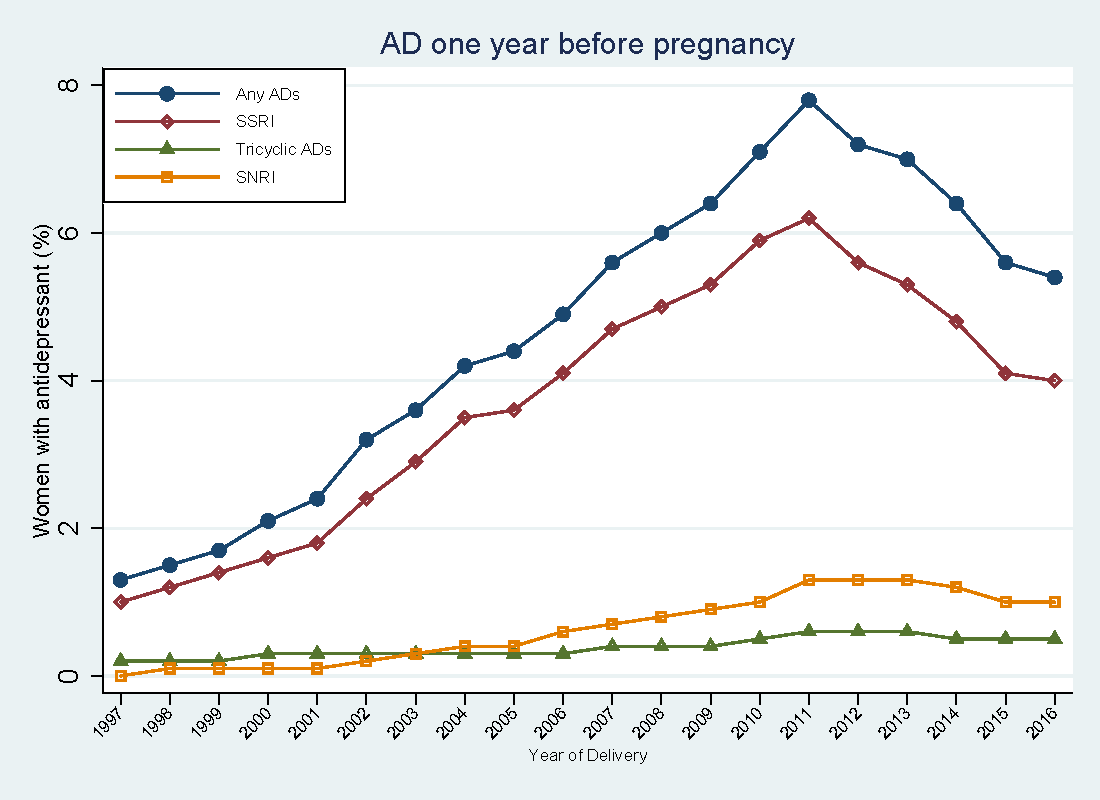


Supplementary Figure 2. Trends in antidepressant prescriptions including types (upper panel) of and specific AD (bottom panel) in one year after pregnancy in women who gave birth between 1997 and 2016 in Denmark


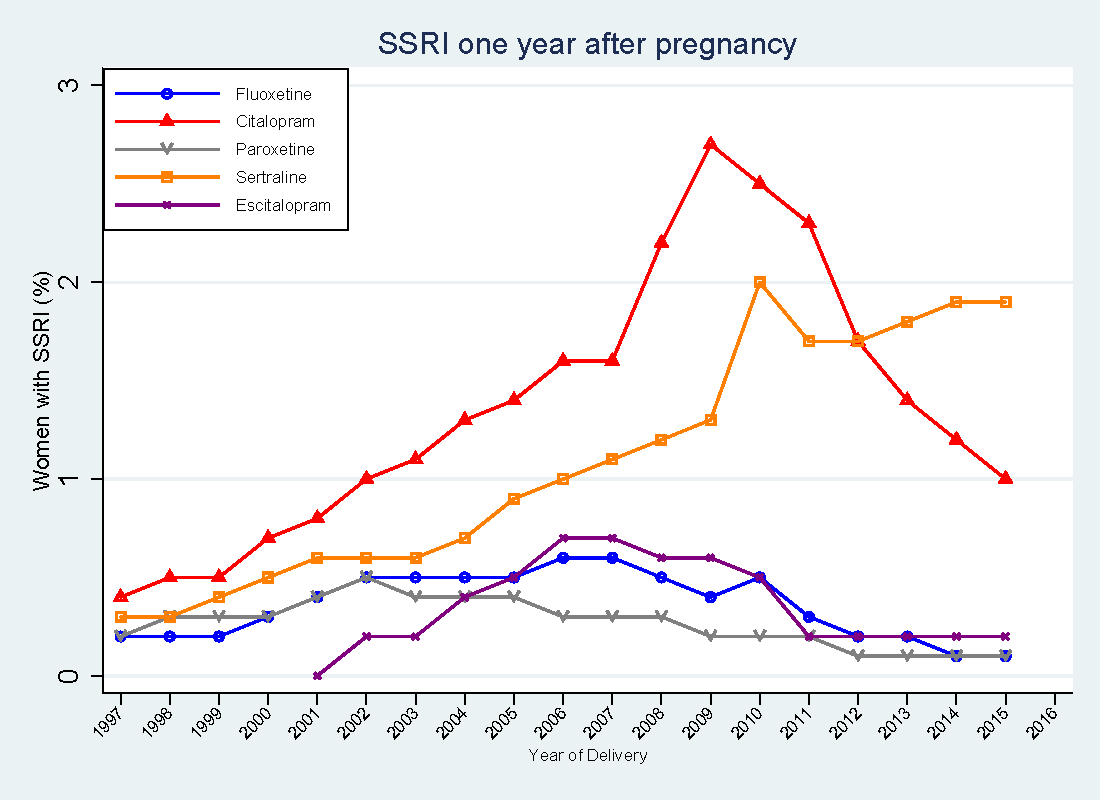


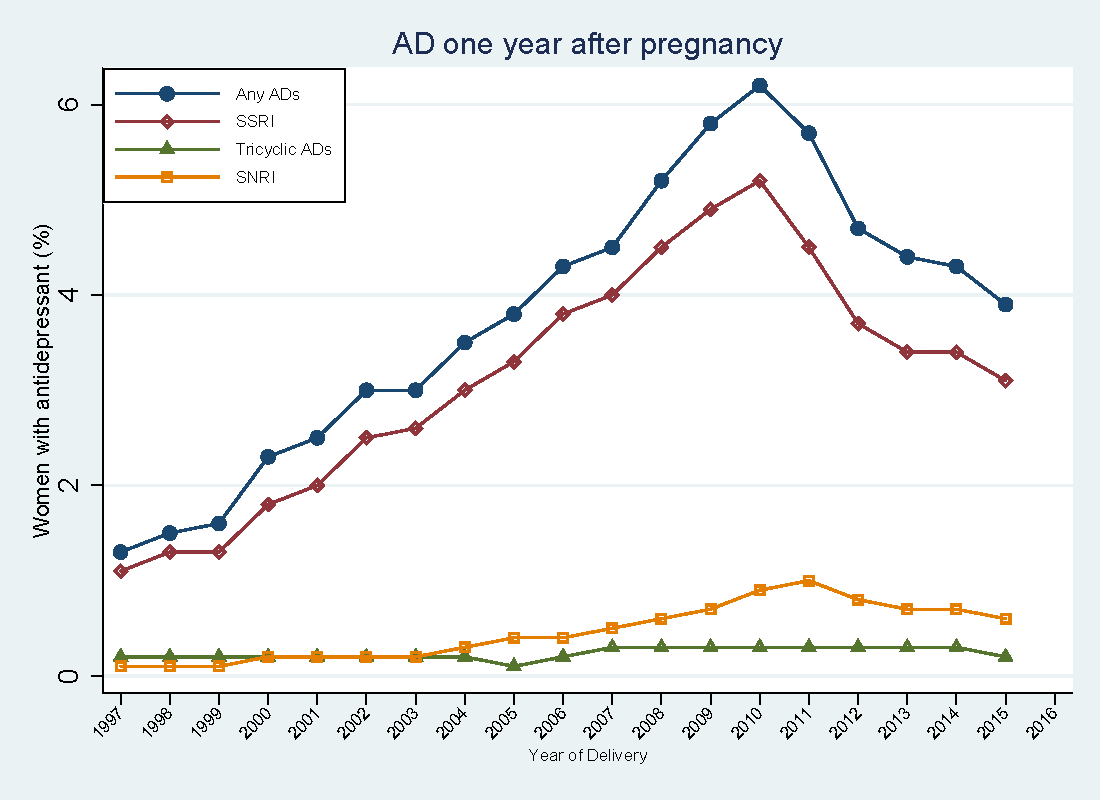


Supplementary Figure 3. Trends in antidepressant prescriptions in one year before pregnancy among women who gave birth in Denmark between 1997 and 2016 according to the age of the pregnant women (upper panel) and the history of psychiatric disorders before birth (bottom panel)


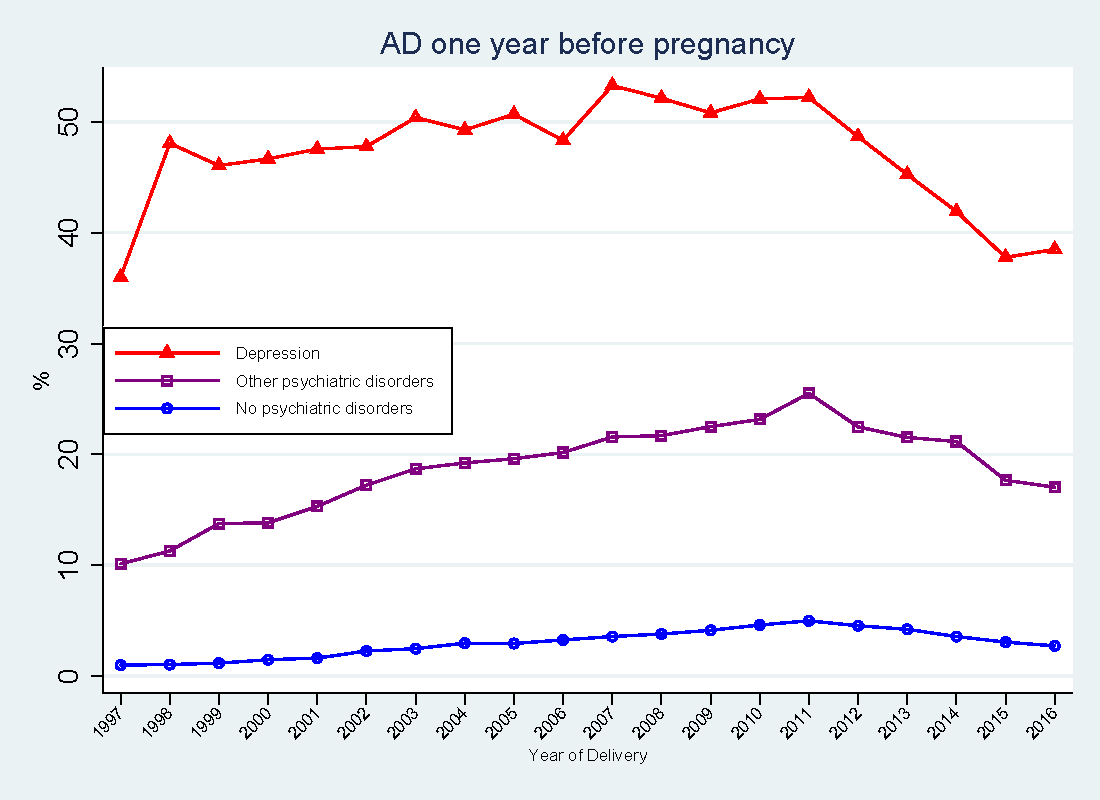


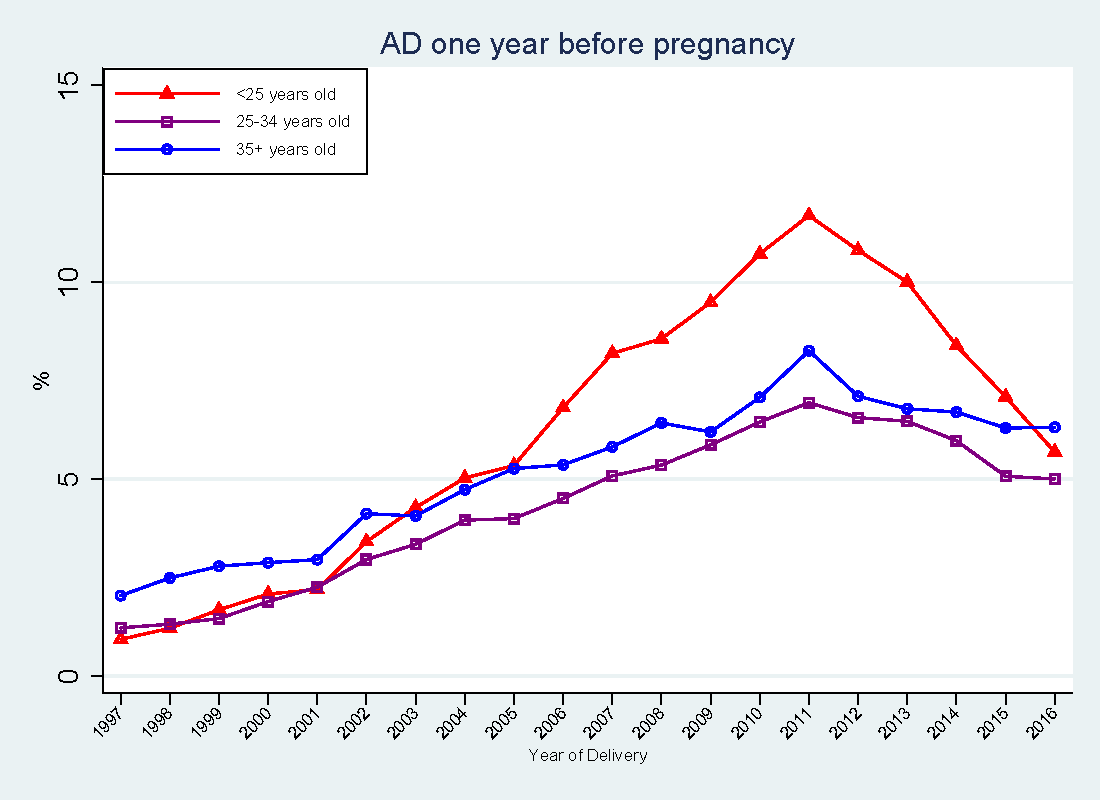


Supplementary Figure 4. Trends in antidepressant prescriptions in one year after pregnancy among women who gave birth in Denmark between 1997 and 2016 according to the age of the pregnant women (upper panel) and the history of psychiatric disorders before birth (bottom panel)


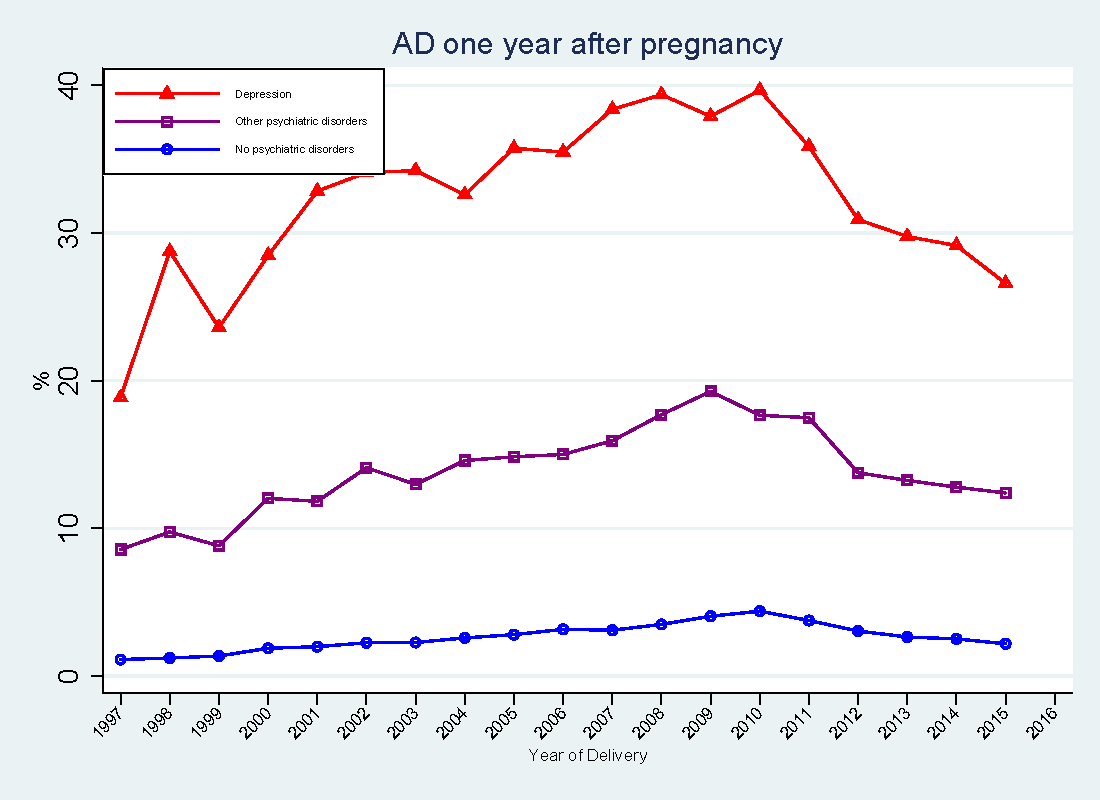


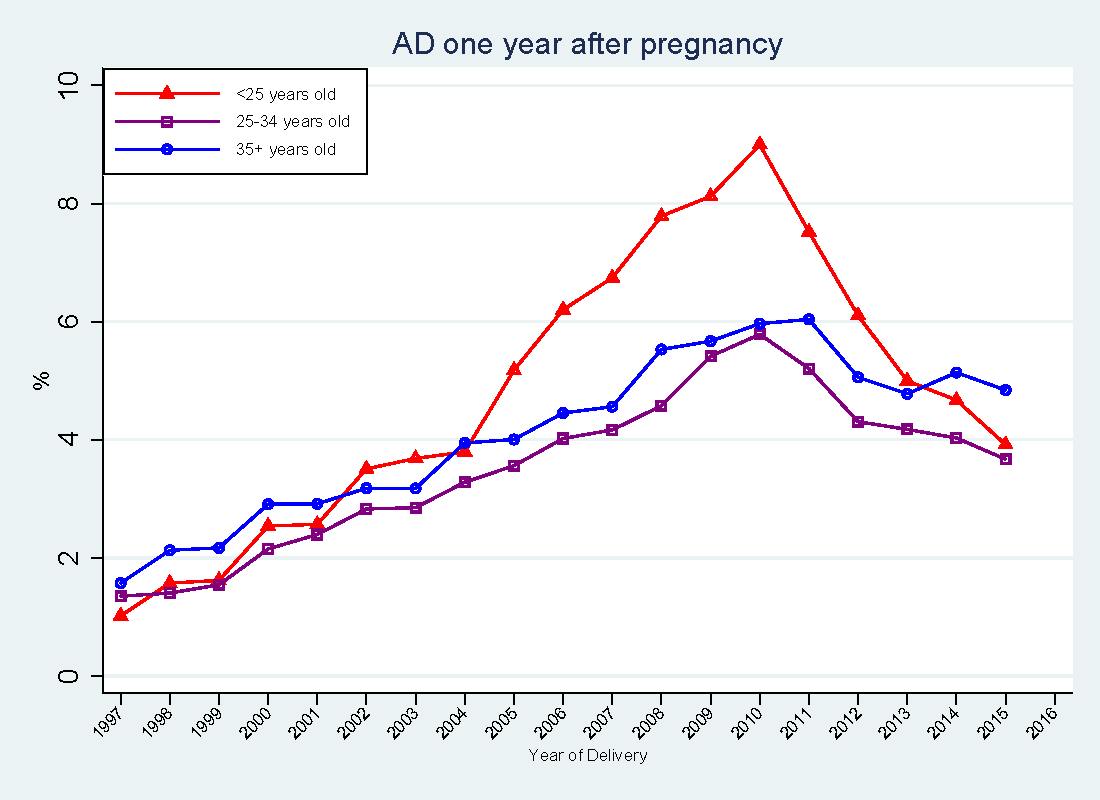


**Supplementary tables**

| Supplementary Table 1. Anatomical therapeutic chemical (ATC) classification codes for antidepressants (ADs) | |
| --- | --- |
| Class of antidepressant | ATC codes |
| All antidepressants | N06A |
| Tricyclic ADs (TCA/non-selective monoamine reuptake inhibitors) | N06AA |
| Selective serotonin reuptake inhibitors (SSRIs) | N06AB |
| Zimeldine | N06AB02 |
| Fluoxetine | N06AB03 |
| Citalopram | N06AB04 |
| Paroxetine | N06AB05 |
| Sertraline | N06AB06 |
| Alaproclate | N06AB07 |
| Fluvoxamine | N06AB08 |
| Etoperidone | N06AB09 |
| Escitalopram | N06AB10 |
| Serotonin and norepinephrine reuptake inhibitors (SNRIs) | N06AX16, N06AX17, N06AX21, N06AX23 |
| Other ADs | Other ATC codes in N06AX after excluding SNRIs, N06AF, N06AG |
| Monoamine oxidase inhibitors, non-selective | N06AF |
| Monoamine oxidase A inhibitors | N06AG |
| Norepinephrine and dopamine reuptake inhibitors (NDRIs) | N06AX12 |

| Supplementary table 2. ICD codes for depression and other psychiatric disorders | | |
| --- | --- | --- |
| Psychiatric disorders | ICD 8 | ICD 10 |
| Depression | 29609, 29629, 29699, 29809, 30049, 30019 | F32-F33 |
| Other psychiatric disorders | 290-315 excluding the codes for depression | F00-F99 excluding the codes for depression |

| Supplementary table 3: Age distribution of pregnant women at time of delivery in the study population | | | | | | | | |
| --- | --- | --- | --- | --- | --- | --- | --- | --- |
| Year of delivery | Age of women at time of delivery | | | | | | | |
|  | <25 years | |  | 25-34 years | |  | 35+ years | |
|  | No. | % |  | No. | % |  | No. | % |
| 1997 | 11,204 | 17.0 |  | 46,315 | 70.3 |  | 8,337 | 12.7 |
| 1998 | 10,504 | 16.2 |  | 45,555 | 70.5 |  | 8,601 | 13.3 |
| 1999 | 10,073 | 15.6 |  | 45,379 | 70.2 |  | 9,223 | 14.3 |
| 2000 | 9,612 | 14.6 |  | 46,376 | 70.6 |  | 9,663 | 14.7 |
| 2001 | 9,078 | 14.2 |  | 44,854 | 70.1 |  | 10,020 | 15.7 |
| 2002 | 8,299 | 13.3 |  | 43,731 | 69.8 |  | 10,603 | 16.9 |
| 2003 | 7,711 | 12.2 |  | 44,501 | 70.5 |  | 10,925 | 17.3 |
| 2004 | 7,302 | 11.6 |  | 44,939 | 71.1 |  | 10,924 | 17.3 |
| 2005 | 7,071 | 11.2 |  | 44,609 | 70.9 |  | 11,239 | 17.9 |
| 2006 | 7,160 | 11.3 |  | 44,893 | 70.6 |  | 11,544 | 18.2 |
| 2007 | 7,104 | 11.3 |  | 43,519 | 69.2 |  | 12,238 | 19.5 |
| 2008 | 7,846 | 12.3 |  | 43,224 | 67.9 |  | 12,588 | 19.8 |
| 2009 | 7,522 | 12.2 |  | 41,512 | 67.4 |  | 12,522 | 20.3 |
| 2010 | 7,768 | 12.5 |  | 41,453 | 66.6 |  | 12,990 | 20.9 |
| 2011 | 7,208 | 12.5 |  | 38,118 | 66.1 |  | 12,322 | 21.4 |
| 2012 | 7,176 | 12.6 |  | 37,446 | 65.9 |  | 12,161 | 21.4 |
| 2013 | 6,825 | 12.5 |  | 36,118 | 66.0 |  | 11,743 | 21.5 |
| 2014 | 6,830 | 12.2 |  | 36,932 | 66.1 |  | 12,070 | 21.6 |
| 2015 | 6,868 | 12.0 |  | 38,286 | 66.9 |  | 12,084 | 21.1 |
| 2016 | 7,124 | 12.0 |  | 40,055 | 67.3 |  | 12,336 | 20.7 |

| Supplementary table 4: Diagnosis of depression and other psychiatric disorders before birth according to the year of delivery | | | | | | | | |
| --- | --- | --- | --- | --- | --- | --- | --- | --- |
|  | Diagnosis of psychiatric disorder before birth | | | | | | | |
| Year of delivery | Depression |  |  | Other psychiatric disorders | |  | No psychiatric disorders | |
|  | No. | % |  | No. | % |  | No. | % |
| 1997 | 175 | 0.3 |  | 1,483 | 2.3 |  | 64,198 | 97.5 |
| 1998 | 233 | 0.4 |  | 1,641 | 2.5 |  | 62,786 | 97.1 |
| 1999 | 267 | 0.4 |  | 1,782 | 2.8 |  | 62,626 | 96.8 |
| 2000 | 330 | 0.5 |  | 2,012 | 3.1 |  | 63,309 | 96.4 |
| 2001 | 408 | 0.6 |  | 2,131 | 3.3 |  | 61,413 | 96.0 |
| 2002 | 563 | 0.9 |  | 2,340 | 3.7 |  | 59,730 | 95.4 |
| 2003 | 631 | 1.0 |  | 2,520 | 4.0 |  | 59,986 | 95.0 |
| 2004 | 773 | 1.2 |  | 2,700 | 4.3 |  | 59,692 | 94.5 |
| 2005 | 870 | 1.4 |  | 2,984 | 4.7 |  | 59,065 | 93.9 |
| 2006 | 1,113 | 1.8 |  | 3,353 | 5.3 |  | 59,131 | 93.0 |
| 2007 | 1,261 | 2.0 |  | 3,584 | 5.7 |  | 58,016 | 92.3 |
| 2008 | 1,473 | 2.3 |  | 3,786 | 5.9 |  | 58,399 | 91.7 |
| 2009 | 1,519 | 2.5 |  | 3,725 | 6.1 |  | 56,312 | 91.5 |
| 2010 | 1,709 | 2.7 |  | 4,078 | 6.6 |  | 56,424 | 90.7 |
| 2011 | 1,762 | 3.1 |  | 3,944 | 6.8 |  | 51,942 | 90.1 |
| 2012 | 1,818 | 3.2 |  | 4,040 | 7.1 |  | 50,925 | 89.7 |
| 2013 | 1,982 | 3.6 |  | 4,070 | 7.4 |  | 48,634 | 88.9 |
| 2014 | 2,140 | 3.8 |  | 4,458 | 8.0 |  | 49,234 | 88.2 |
| 2015 | 2,181 | 3.8 |  | 4,713 | 8.2 |  | 50,344 | 88.0 |
| 2016 | 2,351 | 4.0 |  | 5,126 | 8.6 |  | 52,038 | 87.4 |
